# Supplementary material for: Machine Learning Accelerates Crystallization for Structure Determination
Source: Angew Chem Int Ed Engl. 2026 May 4;65(25):e1218503. doi: 10.1002/anie.1218503 (PMC13266958; doi:10.1002/anie.1218503)

checkCIF/PLATON report

Structure factors have been supplied for datablock(s) lcz054

THIS REPORT IS FOR GUIDANCE ONLY. IF USED AS PART OF A REVIEW PROCEDURE FOR PUBLICATION, IT SHOULD NOT REPLACE THE EXPERTISE OF AN EXPERIENCED CRYSTALLOGRAPHIC REFEREE.

No syntax errors found. CIF dictionary Interpreting this report

Datablock: lcz054

Bond precision:	C-C = 0.0121 A		Wavelength=1.54184
Cell:	a=11.9194(3)	b=12.6845(3)	c=24.4397(6)
	alpha=95.602(2)	beta=92.165(2)	gamma=115.154(2)
Temperature:	150 K		

	Calculated	Reported
Volume	3315.78(15)	3315.78(15)
Space group	P -1	P -1
Hall group	-P 1	-P 1
Moiety formula	C35 H17 Ag3 F18 N6 O2, C15 H3 Ag3 F18 N6 [+ solvent]	C15 H3 Ag3 F18 N6, 0.5(C20 H14 O2)
Sum formula	C50 H20 Ag6 F36 N12 O2 [+ solvent]	C25 H10 Ag3 F18 N6 O
Mr	2152.00	1076.00
Dx,g cm-3	2.155	2.155
Z	2	4
Mu (mm-1)	15.340	15.340
F000	2052.0	2052.0
F000’	2062.06	
h,k,lmax	15,16,30	15,16,30
Nref	14172	13319
Tmin,Tmax	0.198,0.216	0.100,1.000
Tmin’	0.037	

```
Correction method= # Reported T Limits: Tmin=0.100 Tmax=1.000
AbsCorr = MULTI-SCAN

Data completeness= 0.940          Theta(max)= 77.972

R(reflections)= 0.0671( 11457)          wR2(reflections)=
                                        0.1857( 13319)
S = 1.055                   Npar= 977
```

The following ALERTS were generated. Each ALERT has the format
test-name_ALERT_alert-type_alert-level.
Click on the hyperlinks for more details of the test.

Alert level C

```
PLAT213_ALERT_2_C Atom F012           has ADP max/min Ratio .....        3.2 prolat
PLAT213_ALERT_2_C Atom F01I           has ADP max/min Ratio .....        3.2 prolat
PLAT213_ALERT_2_C Atom F01L           has ADP max/min Ratio .....        3.2 prolat
PLAT213_ALERT_2_C Atom F01O           has ADP max/min Ratio .....        3.2 prolat
PLAT213_ALERT_2_C Atom F02E           has ADP max/min Ratio .....        3.1 prolat
PLAT230_ALERT_2_C Hirshfeld Test Diff for    F023     --C028     .       5.2 s.u.
PLAT234_ALERT_4_C Large Hirshfeld Difference F02E     --C02J     .      0.19 Ang.
PLAT234_ALERT_4_C Large Hirshfeld Difference F02Q     --C02H     .      0.19 Ang.
PLAT234_ALERT_4_C Large Hirshfeld Difference F5A      --C1       .      0.16 Ang.
PLAT342_ALERT_3_C Low Bond Precision on  C-C Bonds ..............    0.01213 Ang.
PLAT906_ALERT_3_C Large K Value in the Analysis of Variance ......     4.846 Check
PLAT906_ALERT_3_C Large K Value in the Analysis of Variance ......     2.103 Check
PLAT911_ALERT_3_C Missing FCF Refl Between Thmin & STh/L=    0.600        99 Report
                1  0  0,  -7  3  0,  -1  7  0,  -4-11  1,   1 -7  1,   7 -3  1,
                1  0  1,  10  5  1,  -4-11  2,   1  1  2, -10 14  2,  -5-10  3,
               -5 -2  3,  -1 -2  3, -11 13  3, -10 14  3,  -4-11  4,  -7 -8  4,
               -6 -8  4,  -6 -3  4,   9  6  4,   8  7  4,  -6 -8  5,  -8 -7  5,
               -8 -6  5,  -7 -6  5,  -5 -1  6,  -3  0  6,  -6  1  6,  -1  6  6,
                   (   69 More Missing: see the .ckf listing file)
PLAT971_ALERT_2_C Check Calcd Resid. Dens.  0.95Ang From Ag01         2.09 eA-3
PLAT971_ALERT_2_C Check Calcd Resid. Dens.  0.98Ang From Ag01         2.00 eA-3
PLAT971_ALERT_2_C Check Calcd Resid. Dens.  1.05Ang From Ag06         1.92 eA-3
PLAT971_ALERT_2_C Check Calcd Resid. Dens.  0.88Ang From Ag04         1.91 eA-3
PLAT971_ALERT_2_C Check Calcd Resid. Dens.  1.04Ang From Ag03         1.89 eA-3
PLAT971_ALERT_2_C Check Calcd Resid. Dens.  1.11Ang From Ag04         1.80 eA-3
PLAT971_ALERT_2_C Check Calcd Resid. Dens.  0.99Ang From Ag02         1.77 eA-3
PLAT971_ALERT_2_C Check Calcd Resid. Dens.  1.00Ang From Ag05         1.74 eA-3
PLAT971_ALERT_2_C Check Calcd Resid. Dens.  0.81Ang From Ag02         1.72 eA-3
PLAT971_ALERT_2_C Check Calcd Resid. Dens.  0.94Ang From Ag03         1.71 eA-3
PLAT971_ALERT_2_C Check Calcd Resid. Dens.  1.05Ang From F02Q         1.60 eA-3
PLAT971_ALERT_2_C Check Calcd Resid. Dens.  1.01Ang From Ag05         1.56 eA-3
PLAT971_ALERT_2_C Check Calcd Resid. Dens.  1.19Ang From F01L         1.54 eA-3
PLAT971_ALERT_2_C Check Calcd Resid. Dens.  1.22Ang From F017         1.54 eA-3
```

Alert level G

```
PLAT002_ALERT_2_G Number of Distance or Angle Restraints on AtSite          7 Note
PLAT003_ALERT_2_G Number of Uiso or U(i,j)  Restrained non-H-Atoms         10 Report
```

```
PLAT041_ALERT_1_G Calc. and Reported SumFormula    Strings  Differ     Please Check
              Calc: C50 H20 Ag6 F36 N12 O2
              Rep.: C25 H10 Ag3 F18 N6 O
PLAT042_ALERT_1_G Calc. and Reported MoietyFormula Strings  Differ     Please Check
              Calc: C35 H17 Ag3 F18 N6 O2, C15 H3 Ag3 F18 N6
              Rep.: C15 H3 Ag3 F18 N6, 0.5(C20 H14 O2)
PLAT045_ALERT_1_G Calculated and Reported Z Differ by a Factor ...      0.500 Check
PLAT072_ALERT_2_G SHELXL First  Parameter in WGHT  Unusually Large       0.11 Report
PLAT083_ALERT_2_G SHELXL Second Parameter in WGHT  Unusually Large      15.67 Why ?
PLAT154_ALERT_1_G The s.u.'s on the Cell Angles are Equal ..(Note)      0.002 Degree
PLAT171_ALERT_4_G The CIF-Embedded .res File Contains EADP Records          1 Report
PLAT172_ALERT_4_G The CIF-Embedded .res File Contains DFIX Records          3 Report
PLAT176_ALERT_4_G The CIF-Embedded .res File Contains SADI Records          3 Report
PLAT186_ALERT_4_G The CIF-Embedded .res File Contains ISOR Records          5 Report
PLAT242_ALERT_2_G Low    'MainMol' Ueq as Compared to Neighbors of       C01M Check
PLAT242_ALERT_2_G Low    'MainMol' Ueq as Compared to Neighbors of       C02F Check
PLAT242_ALERT_2_G Low    'MainMol' Ueq as Compared to Neighbors of       C02J Check
PLAT242_ALERT_2_G Low    'MainMol' Ueq as Compared to Neighbors of       C02T Check
PLAT242_ALERT_2_G Low    'MainMol' Ueq as Compared to Neighbors of       C02U Check
PLAT242_ALERT_2_G Low    'MainMol' Ueq as Compared to Neighbors of       C02W Check
PLAT242_ALERT_2_G Low    'MainMol' Ueq as Compared to Neighbors of         C1 Check
PLAT242_ALERT_2_G Low    'MainMol' Ueq as Compared to Neighbors of       C027 Check
PLAT242_ALERT_2_G Low    'MainMol' Ueq as Compared to Neighbors of       C028 Check
PLAT242_ALERT_2_G Low    'MainMol' Ueq as Compared to Neighbors of       C029 Check
PLAT242_ALERT_2_G Low    'MainMol' Ueq as Compared to Neighbors of       C02H Check
PLAT242_ALERT_2_G Low    'MainMol' Ueq as Compared to Neighbors of       C02P Check
PLAT302_ALERT_4_G Anion/Solvent/Minor-Residue Disorder (Resd    2)         7% Note
PLAT434_ALERT_2_G Short Inter HL..HL Contact F00R     ..F01L     .       2.80 Ang.
                                                     x,y,z  =      1_555 Check
PLAT434_ALERT_2_G Short Inter HL..HL Contact F01Q     ..F01Q     .       2.81 Ang.
                                                1-x,1-y,-z  =      2_665 Check
PLAT434_ALERT_2_G Short Inter HL..HL Contact F01W     ..F01W     .       2.74 Ang.
                                                1-x,2-y,-z  =      2_675 Check
PLAT434_ALERT_2_G Short Inter HL..HL Contact F023     ..F02E     .       2.70 Ang.
                                                     x,y,z  =      1_555 Check
PLAT605_ALERT_4_G Largest Solvent Accessible VOID in the Structure        198 A**3
PLAT720_ALERT_4_G Number of Unusual/Non-Standard Labels ..........        122 Note
              Ag01    Ag02    Ag03    Ag04    Ag05    Ag06    O007    O008
              F009    N00A    N00B    F00C    N00D    N00E    F00F    N00G
              N00H    N00I    F00J    N00K    N00L    F00M    N00N    N00O
              F00P    N00Q    F00R    F00S    F00T    F00U    F00V    C00W
              F00X    F00Y    C00Z    F010    C011    F012    C013    H013
              C014    H014    F015    C016    F017    C018    C019    F01A
              C01B    C01C    H01C    F01D    C01E    H01E    C01F    F01G
              C01H    H01H    F01I    F01J    C01K    H01K    F01L    C01M
              C01N    F01O    F01P    F01Q    C01R    H01R    C01S    H01S
              F01T    C01U    C01V    H01V    F01W    C01X    C01Y    H01Y
              C01Z    C020    C021    C022    F023    C024    H024    C025
              H025    C026    C027    C028    C029    C02A    H02A    C02B
              H02B    C02C    H02C    C02D    H02D    F02E    C02F    C02G
              H02G    C02H    F02I    C02J    C02K    C02L    H02L    F02M
              C02N    H02N    C02O    C02P    F02Q    C02T    C02U    C02V
              H02V    C02W
PLAT860_ALERT_3_G Number of Least-Squares Restraints ............          66 Note
PLAT868_ALERT_4_G ALERTS Due to the Use of _smtbx_masks Suppressed          ! Info
PLAT883_ALERT_1_G Absent Datum for _atom_sites_solution_primary ..     Please Do !
PLAT910_ALERT_3_G Missing FCF Reflection(s) Below Theta(Min)[Deg]=       3.88 Note
```

```
               0  0  1,   0  0  2,
PLAT912_ALERT_4_G Missing # of FCF Reflections Above STh/L=  0.600        752 Note
PLAT913_ALERT_3_G Missing # of Very Strong Reflections in FCF ....          1 Note
              -1 -2  3,
PLAT933_ALERT_2_G Number of HKL-OMIT Records in Embedded .res File         12 Note
              -8 -7  5,  -8 -6  5,  -7 -9  4,  -7 -9  5,  -7 -8  4,  -6-10  4,
              -6-10  5,  -5-11  4,  -4 -9  9,  -4 -9 15,  -3-10 15,   4 12  0,
PLAT941_ALERT_3_G Average HKL Measurement Multiplicity ..........        2.1 Low
PLAT969_ALERT_5_G The 'Henn et al.' R-Factor-gap value ..........      3.769 Note
             Predicted wR2: Based on SigI**2  4.93 or SHELX Weight 17.59
PLAT978_ALERT_2_G Number C-C Bonds with Positive Residual Density.          0 Info
```

```
   0 ALERT level A = Most likely a serious problem - resolve or explain
   0 ALERT level B = A potentially serious problem, consider carefully
  27 ALERT level C = Check. Ensure it is not caused by an omission or oversight
  41 ALERT level G = General information/check it is not something unexpected

   5 ALERT type 1 CIF construction/syntax error, inconsistent or missing data
  42 ALERT type 2 Indicator that the structure model may be wrong or deficient
   8 ALERT type 3 Indicator that the structure quality may be low
  12 ALERT type 4 Improvement, methodology, query or suggestion
   1 ALERT type 5 Informative message, check
```

Validation response form

Please find below a validation response form (VRF) that can be filled in and pasted into your CIF.

```
# start Validation Reply Form
_vrf_PLAT213_lcz054
;
PROBLEM: Atom F012            has ADP max/min Ratio .....        3.2 prolat
RESPONSE: ...
;
_vrf_PLAT230_lcz054
;
PROBLEM: Hirshfeld Test Diff for    F023     --C028     .        5.2 s.u.
RESPONSE: ...
;
_vrf_PLAT234_lcz054
;
PROBLEM: Large Hirshfeld Difference F02E     --C02J     .       0.19 Ang.
RESPONSE: ...
;
_vrf_PLAT342_lcz054
;
PROBLEM: Low Bond Precision on  C-C Bonds ..............    0.01213 Ang.
RESPONSE: ...
;
_vrf_PLAT906_lcz054
;
PROBLEM: Large K Value in the Analysis of Variance ......      4.846 Check
RESPONSE: ...
;
_vrf_PLAT911_lcz054
;
```

```
PROBLEM: Missing FCF Refl Between Thmin & STh/L=    0.600         99 Report
RESPONSE: ...
;
_vrf_PLAT971_lcz054
;
PROBLEM: Check Calcd Resid. Dens.  0.95Ang From Ag01           2.09 eA-3
RESPONSE: ...
;
# end Validation Reply Form
```

It is advisable to attempt to resolve as many as possible of the alerts in all categories. Often the minor alerts point to easily fixed oversights, errors and omissions in your CIF or refinement strategy, so attention to these fine details can be worthwhile. It is up to the individual to critically assess their own results and, if necessary, seek expert advice.

PLATON version of 17/09/2025; check.def file version of 17/09/2025

---

duplicate check

No duplication found

Datablock lcz054 - ellipsoid plot

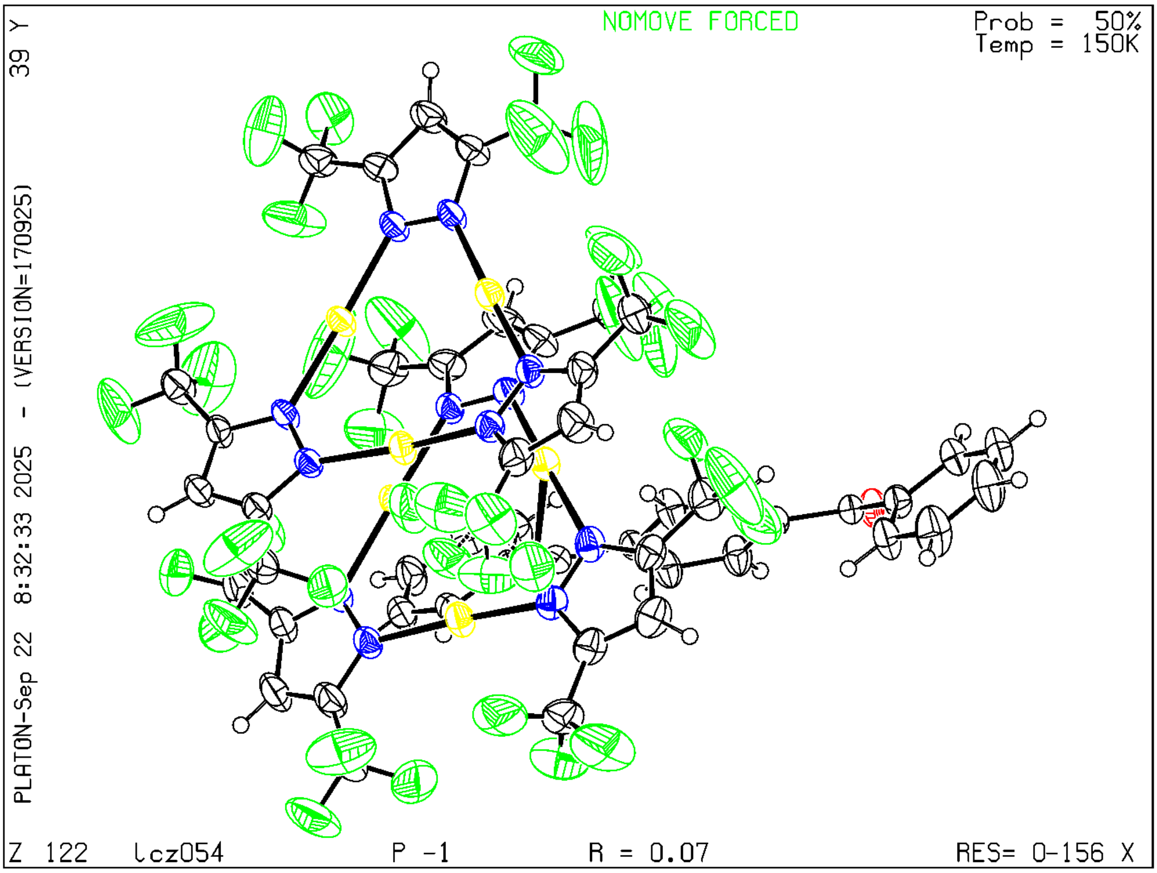

Supplement: Supplementary file 2 — Supporting File 2: anie72462‐sup‐0002‐DataFile.zip. [file ANIE-65-e1218503-s002.zip › supplementary_materials_crystallographic_data submit.zip!/cif_and_checkcif(1-114)/Ag3Pz3_54/Ag3Pz3_54_checkcif.pdf]
